# Quantitative comparisons of select cultured and uncultured microbial populations in the rumen of cattle fed different diets

**DOI:** 10.1186/2049-1891-3-28

**Published:** 2012-09-07

**Authors:** Minseok Kim, Zhongtang Yu

**Affiliations:** 1 Department of Animal Sciences, The Ohio State University, 2029 Fyffe Road, Columbus, OH 43210, USA

**Keywords:** 16S rRNA gene, Real-time PCR, Rumen, Uncultured bacteria

## Abstract

**Background:**

The number and diversity of uncultured ruminal bacterial and archaeal species revealed by 16S rRNA gene (*rrs*) sequences greatly exceeds that of cultured bacteria and archaea. However, the significance of uncultured microbes remains undetermined. The objective of this study was to assess the numeric importance of select uncultured bacteria and cultured bacteria and the impact of diets and microenvironments within cow rumen in a comparative manner.

**Results:**

Liquid and adherent fractions were obtained from the rumen of Jersey cattle fed hay alone and Holstein cattle fed hay plus grain. The populations of cultured and uncultured bacteria present in each fraction were quantified using specific real-time PCR assays. The population of total bacteria was similar between fractions or diets, while total archaea was numerically higher in the hay-fed Jersey cattle than in the hay-grain-fed Holstein cattle. The population of the genus *Prevotella* was about one log smaller than that of total bacteria. The populations of *Fibrobacter succinogenes*, *Ruminococcus flavefaciens*, the genus *Butyrivibrio*, and *R. albus* was at least one log smaller than that of genus *Prevotella*. Four of the six uncultured bacteria quantified were as abundant as *F. succinogenes*, *R. flavefaciens* and the genus *Butyrivibrio*. In addition, the populations of several uncultured bacteria were significantly higher in the adherent fractions than in the liquid fractions. These uncultured bacteria may be associated with fiber degradation.

**Conclusions:**

Some uncultured bacteria are as abundant as those of major cultured bacteria in the rumen. Uncultured bacteria may have important contribution to ruminal fermentation. Population dynamic studies of uncultured bacteria in a comparative manner can help reveal their ecological features and importance to rumen functions.

## Background

A complex ruminal microbiome mediates hydrolysis of polymeric feedstuffs and subsequent fermentation to volatile fatty acids (VFA) that are used as the energy source for ruminant animals [1]. Microbial biomass also constitutes the major sources of protein and B vitamins for the host animals. Being the major contributors to rumen functions, bacteria have been the focus of microbiological studies of the rumen microbiome. Cultivation-based methods were used to investigate ruminal bacteria until the 1980s. As a result, various cultured bacteria were identified, and their functions were determined through physiological studies of model species or strains. Once *rrs* sequences were used to investigate diversity of ruminal bacteria [2], it became evident that cultured ruminal bacteria represent only a small portion of the ruminal bacteriome [3,4]. Kim *et al.* reported that *rrs* sequences obtained from cultured bacteria represent only 7% of all the bacterial sequences of rumen origin [4]. More than 55% of all the bacterial sequences were assigned to groups that could not be classified into any known genus [4]. Therefore, uncultured members of the ruminal bacteriome probably play a greater role in rumen functions than the cultured peers.

Frequencies of *rrs* sequences are often used to infer the abundance and population dynamics of the uncultured bacteria represented. However, PCR using universal primers is well documented to have amplification bias [5]. As such, sequence frequency does not necessarily reflect the relative abundance of the bacterium represented, or the importance or weight to rumen function. In a previous study [6], specific real-time PCR assays were developed to accurately determine the distribution and population sizes of uncultured bacteria in the rumen of sheep. Some uncultured bacteria had abundance comparable to that of several cultured bacteria that are perceived as major bacteria in the rumen. We hypothesize that this also holds true for the rumen of cattle. To test this hypothesis, real-time PCR assays were used to quantify the populations of select cultured and uncultured bacteria in the rumen of cattle fed different diets.

## Methods

### Sample collection, fractionation and DNA extraction

Two cannulated Jersey cattle were fed only hay composed mainly of Timothy grass (designated as H), and two cannulated Holstein cattle were fed a mixture consisting of 14% alfalfa forage, 42% corn silage, 6% cottonseed, and 38% grains (designated as C) as described previously [7]. The four cannulated cattle were fed twice daily (early morning and late afternoon) and allowed to adapt to their respective diets for more than 3 weeks prior to rumen sampling. Rumen digesta samples were collected from the four cannulated cattle approximately 6 h after the morning feeding. Bacteria present in the liquid (Lq) and the adherent (Ad) fractions were recovered as described previously [7]. Eight fraction samples (2 cattle × 2 diets × 2 fractions) were stored at −80°C prior to DNA extraction. Metagenomic DNA was extracted from each sample as described previously [8].

### Real-time PCR assays

The primers and the PCR conditions used in quantifying each target were the same as those used by Stiverson *et al.*[6]. End-point PCR was performed to amplify the standards for *Fibrobacter succinogenes*, *Ruminococcus albus*, and *Prevotella ruminicola* from the genomic DNA of respective strains using the 27 F (5′- AGA GTT TGA TCM TGG CTC AG-3′) and the 1525R (5′- AAG GAG GTG WTC CAR CC-3′) primers on a PTC-100 thermocycler (MJ Research, Waltham, MA). On the other hand, a composite sample of the eight metagenomic DNA samples at equal amount was used to prepare sample-derived standards for total bacteria, total archaea, *Butyrivibrio*, *Prevotella*, *Ruminobacter amylophilus*, *Ruminococcus flavefaciens*, *Selenomonas ruminantium*, and the six uncultured bacteria by end-point PCR. The six uncultured bacterial *rrs* clones, Ad-C1-74-3, Lq-C2-16-3, Lq-C2-58-2, Ad-H1-14-1, Ad-H1-75-1 and Ad-H2-90-2, were recovered from sheep fed two different diets [6,9]. The sample-derived standards were used to reduce bias that may result from sequence variation within total bacteria, total archaea, *Butyrivibrio*, or *Prevotella* as described previously [10]. The sample-derived standards for *R. amylophilus*, *R. flavefaciens* and *S. ruminantium* were used because their purified genomic DNAs were not available. These sample-derived standards were amplified from the composite DNA sample using the respective primer sets as described previously [6] and then purified using a QIAquick PCR purification kit (QIAGEN). Each standard was serially diluted and the concentration from 10^2^ to 10^8^*rrs* copies per reaction were used in the real-time PCR assays.

Each real-time PCR assay was conducted in three technical replicates (three PCR reactions from the same DNA template) from which the mean was calculated on a Stratagene Mx3000p machine (La Jolla, CA, USA). All the cultured and the uncultured bacteria were quantified using SYBR green I (Molecular Probes) except for total bacteria that were quantified using the TaqMan assay using the same conditions as reported by Stiverson *et al.*[6]. The mean was also calculated from the two biological replicates (two cattle fed the same diet) and the three technical replicates of each fraction recovered from each diet.

### Statistical analysis

The abundance (*rrs* copies per μg metagenomic DNA) of the cultured and the uncultured bacteria were compared among the four fractions using one-way analysis of variance (ANOVA) as implemented in SAS 9.1 (SAS Institute Inc., Cary, NC). Tukey’s test was used to analyze difference in bacterial abundance among the four fractions. Significance was declared at *P ≤* 0.05.

## Results and discussion

### Quantification of populations of total bacteria and total archaea

Total bacterial populations ranging from 1.71 × 10^8^ to 5.19 × 10^8^*rrs* copies/μg DNA did not differ between the liquid (Lq) and the adherent (Ad) fractions or between the Jersey cattle fed hay only (H) and the Holstein cattle fed corn silage and corn (C) (Table 1). However, total bacterial populations tended (*P* < 0.1) to be higher in the C-fed Holstein cattle than in the H-fed Jersey. Total archaeal populations ranging from 7.22 × 10^2^ to 3.16 × 10^4^*rrs* copies/μg DNA did not differ among the four fractions but numerically higher in the H-fed sheep than in the C-fed sheep (Table 1). It appears that the abundance of total archaea is affected by the amount of forage in the diet. This result corroborates the previous finding that more methane is produced by animals fed diets high in forage than by animals fed diets high in grain [11]. However, it remains to be determined if the animal breeds have any effects on rumen bacteria and archaea.

**Table 1:** Copy numbers, and relative abundance of total bacteria in each fraction^*^

**Taxon**	**Lq-H (Jersey)**	**Lq-C (Holstein)**	**Ad-H (Jersey)**	**Ad-C (Holstein)**	***P *value**
Total bacteria	1.71×10^8^ ± 1.00×10^8^	5.19×10^8^ ± 5.65×10^7^	1.86×10^8^ ± 2.85×10^7^	4.98×10^8^ ± 5.00×10^7^	0.031
Total archaea	2.62×10^4^ ± 1.87×10^4^	7.22×10^2^ ± 7.60×10^1^	3.16×10^4^ ± 3.75×10^3^	3.43×10^3^ ± 2.07×10^3^	0.183
*F. succinogenes*	3.32×10^6^ ± 1.32×10^6^	3.56×10^6^ ± 4.50×10^5^	5.76×10^6^ ± 1.15×10^6^	5.20×10^6^ ± 1.75×10^6^	0.503
	(2.27 ± 0.56%)^ab^	(0.68 ± 0.01%)^b^	(3.09 ± 0.14%)^a^	(1.09% ± 0.46)^ab^	(0.031)
*R. albus*	7.59×10^4^ ± 4.41×10^4^^(b)^	5.31×10^5^ ± 1.90×10^4^^(a)^	3.60×10^5^ ± 1.44×10^5^^(ab)^	5.87×10^5^ ± 4.15×10^4^^(a)^	0.033
	(0.05 ± 0.00%)	(0.10 ± 0.01%)	(0.19 ± 0.05%)	(0.12 ± 0.00%)	(0.067)
*R. flavefaciens*	2.56×10^6^ ± 8.24×10^5^^(c)^	8.27×10^6^ ± 4.31×10^5^^(b)^	8.58×10^6^ ± 1.88×10^5^^(b)^	2.07×10^7^ ± 4.10×10^5^^(a)^	<0.001
	(1.86 ± 0.61%)^b^	(1.60 ± 0.09%)^a^	(4.71 ± 0.62%)^a^	(4.20 ± 0.34%)^a^	(0.020)
*Butyrivibrio*	1.91×10^6^ ± 8.65×10^5^	3.46×10^6^ ± 7.65×10^5^	3.56×10^6^ ± 6.95×10^5^	3.43×10^6^ ± 1.20×10^5^	0.374
	(1.25 ± 0.23%)	(0.66 ± 0.08%)	(2.02 ± 0.68%)	(0.70 ± 0.09%)	(0.156)
*P. ruminicola*	1.94×10^5^ ± 1.58×10^5^^(b)^	6.31×10^6^ ± 1.35×10^6^^(a)^	9.40×10^5^ ± 1.91×10^5^^(b)^	3.88×10^6^ ± 6.20×10^5^^(ab)^	0.014
	(0.09 ± 0.04%)^b^	(1.26 ± 0.40%)^a^	(0.50 ± 0.03%)^ab^	(0.78 ± 0.05%)^ab^	(0.059)
*Prevotella*	4.40×10^7^ ± 2.96×10^7^^(b)^	1.88×10^8^ ± 2.60×10^7^^(a)^	5.95×10^7^ ± 5.45×10^6^^(b)^	1.20×10^8^ ± 1.00×10^6^^(ab)^	0.022
	(23.83 ± 3.35%)	(37.19 ± 9.06%)	(33.25 ± 8.01%)	(24.43 ± 2.63%)	(0.458)
*S. ruminantium*	1.33×10^5^ ± 1.08×10^5^	8.05×10^5^ ± 2.57×10^5^	1.82×10^5^ ± 2.15×10^4^	6.51×10^5^ ± 1.68×10^5^	0.098
	(0.06 ± 0.03%)	(0.16 ± 0.07%)	(0.10 ± 0.00%)	(0.13 ± 0.02%)	(0.394)
*R. amylophilus*	8.18×10^4^ ± 3.61×10^4^	8.43×10^5^ ± 3.48×10^5^	1.04×10^5^ ± 5.86×10^4^	3.02×10^4^ ± 2.46×10^4^	0.084
	(0.05 ± 0.01%)	(0.17 ± 0.09%)	(0.05 ± 0.02%)	(0.01 ± 0.00%)	(0.198)
Ad-C1-74-3	7.32×10^4^ ± 4.27×10^4^^(b)^	1.00×10^5^ ± 2.05×10^3^^(b)^	1.86×10^5^ ± 2.95×10^4^^(ab)^	3.16×10^5^ ± 4.75×10^4^^(a)^	0.027
	(0.04 ± 0.00%)	(0.02 ± 0.00%)	(0.10 ± 0.03%)	(0.06 ± 0.02%)	(0.104)
Lq-C2-16-3	4.55×10^5^ ± 6.05×10^4^^(b)^	9.79×10^5^ ± 4.90×10^4^^(b)^	1.42×10^6^ ± 6.50×10^4^^(ab)^	3.24×10^6^ ± 7.75×10^5^^(a)^	0.003
	(0.38 ± 0.18%)	(0.19 ± 0.03%)	(0.79 ± 0.16%)	(0.67 ± 0.22%)	(0.180)
Lq-C2-58-2	1.22×10^6^ ± 7.89×10^5^^(b)^	3.08×10^6^ ± 8.40×10^5^^(b)^	4.35×10^6^ ± 9.50×10^4 (b)^	9.43×10^6^ ± 1.15×10^6^^(a)^	0.008
	(0.68 ± 0.06%)^b^	(0.62 ± 0.23%)^b^	(2.40 ± 0.42%)^a^	(1.89 ± 0.04%)^ab^	(0.015)
Ad-H1-14-1	1.45×10^6^ ± 2.15×10^5 (b)^	2.62×10^6^ ± 2.25×10^5^^(b)^	2.53×10^6^ ± 2.95×10^5 (b)^	6.07×10^6^ ± 5.40×10^5 (a)^	0.003
	(1.18 ± 0.57%)	(0.51 ± 0.01%)	(1.37 ± 0.05%)	(1.22 ± 0.01%)	(0.288)
Ad-H1-75-1	2.22×10^5^ ± 4.30×10^4 (b)^	1.11×10^4^ ± 7.00×10^2 (b)^	8.59×10^5^ ± 2.65×10^4 (a)^	1.61×10^5^ ± 7.36×10^4 (b)^	0.001
	(0.18 ± 0.08%)^ab^	(0.00 ± 0.00%)^b^	(0.48 ± 0.09%)^a^	(0.03 ± 0.02%)^b^	(0.015)
Ad-H2-90-2	9.26×10^5^ ± 6.73×10^5^	2.03×10^6^ ± 2.50×10^5^	1.77×10^6^ ± 2.60×10^5^	3.72×10^6^ ± 6.50×10^5^	0.067
	(0.48 ± 0.11%)	(0.40 ± 0.09%)	(1.00 ± 0.29%)	(0.77 ± 0.21%)	(0.256)

^*^Values represent means ± standard errors.

Note: Values in parentheses indicate relative abundance of total bacteria.

### Quantification of cultured bacteria

The populations of three major cellulolytic bacteria and *Butyrivibrio* spp. were quantified using respective specific real-time PCR assays. The populations of *F. succinogenes*, and *Butyrivibrio* spp. did not differ among the four fractions irrespective of diets or animal breeds. The population of *R. flavefaciens* was significantly higher in the Ad-C fraction than in the other three fractions and significantly lower in the Lq-H fraction than in the other three fractions (Table 1). However, the relative abundance of *F. succinogenes* was significantly higher in the Ad-H fraction than in the Lq-C fraction (Table 1). The population of *R. albus* was significantly lower in the Lq-H fraction than in the Lq-C and the Ad-C fractions (Table 1). Among the three cellulolytic bacteria, the populations of *F. succinogenes* (1.61 × 10^6^ to 9.96 × 10^6^*rrs* copies/μg DNA) and *R. flavefaciens* (2.56 × 10^6^ to 2.07 × 10^7^*rrs* copies/μg DNA) were more abundant than that of *R. albus* (7.59 × 10^4^ to 5.87 × 10^5^) in any of the fractionated samples. This result supports the previous findings that the population of *F. succinogenes* is higher than that of *R. albus *[3,12,13]. However, some studies on the rumen microbiome showed contradictory results [6,14]. In the two latter studies [6,14], *R. albus* was found to be the most predominant among the three cellulolytic species in the rumen. More studies that use the same procedures for metagenomic DNA extraction and real-time PCR assays are needed to verify the predominance of *R. albus* in the rumen in the context of diet and feeding regime. *F. succinogenes* is a predominant cellulolytic species in the rumen of cattle. Although real-time PCR assays showed the relatively high abundance of *F. succinogenes*, no *Fibrobacteres*-like *rrs* sequences were identified from *rrs* clone libraries constructed from the same rumen contents as described previously [7]. The lack of *Fibrobacteres*-like *rrs* sequences seems to be due to the poor efficiency of PCR amplification with universal primers as demonstrated previously [9]. Therefore, *Fibrobacteres*-specific primers are needed to account for the population of *F. succinogenes* when clone libraries, denaturing gradient gel electrophoresis (DGGE) or pyrosequencing are performed in future studies. The population of the genus *Butyrivibrio* was greater than 10^6^*rrs* copies/μg DNA and did not differ among the four fractions (Table 1).

The population of the genus *Prevotella* ranged from 4.40 × 10^7^ to 1.88 × 10^8^*rrs* copies/μg DNA across all the fractions and was significantly higher in the Lq-C fraction than in the Lq-H and the Ad-H fractions (Table 1). The genus *Prevotella* was the most abundant among known ruminal genera, and its relative abundance ranged from 24% to 37% of total bacteria across the four fractions (Table 1). This result supports that *Prevotella* is the most predominant genus in the rumen [3,4]. The relatively high abundance of the genus *Prevotella* in the Ad-H fraction might suggest their involvement in fiber degradation as described previously [15,16]. Both *Selenomonas ruminantium* and *Ruminobacter amylophilus* did not show any significant difference among the four fractions, but *R. amylophilus* tended (*P* < 0.1) to be more abundant in the Lq-C fraction than in the Ad-C fraction (Table 1).

The population of *P. ruminicola*, the major species of genus *Prevotella* in the rumen*,* was significantly higher in the Lq-C fraction than in the Lq-H and the Ad-H fractions (Table 1). The relative abundance of *P. ruminicola* was very low compared to that of genus *Prevotella* (Table 1). The low relative abundance of *P. ruminicola* is consistent with the finding of two previous studies [3,17], but it does not support the majority status of this species in the rumen. This result also suggests the presence of numerous uncultured *Prevotella* strains [12]. Isolation and characterization of uncultured *Prevotella* strains would help characterize as-yet uncultured *Prevotella* strains in future studies.

It should be noted that the abundance of the genus *Prevotella* might have been overestimated because the *Prevotella*-“specific” primers used in this study matched numerous non-*Prevotella rrs* sequences when compared with 13478 sequences of rumen origin [4]. In addition to 811 *Prevotella* sequences, the forward primer matched 582 non-*Prevotella* sequences belonging to genera *Paraprevotella* (21 sequences), *Rikenella* (18 sequences), *Tannerella* (4 sequences), *Paludibacter* (4 sequences), *Bacteroides* (4 sequences), *Barnesiella* (1 sequence), *Hallella* (1 sequence), and unclassified *Bacteroidales* (298 sequences), unclassified *Bacteroidetes* (113 sequences), other genera of family *Prevotellaceae* (77 sequences), unclassified *Porphyromonadaceae* (40 sequences), and unclassified *Clostridiales* (1 sequence). The reverse primer matched 541 *Prevotella* sequences and 59 non-*Prevotella* sequences that belong to other genera of family *Prevotellaceae* (29 sequences), unclassified *Bacteroidales* (6 sequences), *Paraprevotella* (20 sequences), and *Bacteroides* (4 sequences).

The population of *Prevotella* might have been overestimated in other studies due to lack of specificity of the primers used. For example, the forward primers used in the study by Stiverson and Weimer and Weimer *et al. *[3,17] matched 974 *Prevotella* and 1055 non-*Prevotella* sequences collected from the rumen. The 1055 non-*Prevotella* sequences were assigned to unclassified *Bacteroidales* (477 sequences), unclassified *Bacteroidetes* (168 sequences), other genera of family *Prevotellaceae* (140 sequences), unclassified *Porphyromonadaceae* (129 sequences), unclassified *Clostridiales* (1 sequence), *Rikenella* (42 sequences), *Barnesiella* (40 sequences), *Paraprevotella* (37 sequences), *Hallella* (11 sequences), *Tannerella* (4 sequences), *Bacteroides* (4 sequences), *Paludibacter* (1 sequence), and *Alkaliflexus* (1 sequence); while the reverse primer matched 389 *Prevotella* and 88 non-*Prevotella* sequences. The 88 non-*Prevotella* sequences were assigned to other genera of family *Prevotellaceae* (74 sequences), unclassified *Bacteroidales* (6 sequences), and *Hallella* (8 sequences). Therefore, new primers are needed to improve specific quantification of this important genus in the rumen.

### Quantification of uncultured bacteria

The populations of six different uncultured bacteria were quantified using specific real-time PCR assays. Ad-C1-74-3, Lq-C2-16-3 and Lq-C2-58-2 were originally recovered from sheep fed a mixture of corn and hay, whereas Ad-H1-14-1, Ad-H1-75-1 and Ad-H2-90-2 were recovered from sheep fed hay only [6,9]. The populations of Ad-C1-74-3 and Lq-C2-16-3 were significantly higher in the Ad-C fraction than in the Lq-C and the Lq-H fractions (Table 1). Ad-C1-74-3 and Lq-C2-16-3 were assigned to *Anaerovorax *[6] and ‘Unclassified *Ruminococcaceae*’, respectively. Since *Anaerovorax* spp. of non-rumen origin metabolizes amino acids [18], Ad-C1-74-3 may be associated with the degradation of amino acids in the rumen. The population of Lq-C2-58-2 was significantly higher in the Ad-C fraction than in the other fractions (Table 1). Lq-C2-58-2 was assigned to ‘Unclassified *Erysipelotrichaceae*’. The previous study on sheep [6] also showed that the population of Lq-C2-58-2 was the most abundant in the Ad-C fraction. However, the relative abundance of Lq-C2-58-2 was significantly higher in the Ad-H fraction than in the Lq fractions and accounted for more than 2% of total bacteria (Table 1). The Lq-C2-58-2 might be commonly abundant in the adherent fraction of corn-fed ruminants.

The populations of Ad-H1-14-1 and Ad-H2-90-2, which were assigned to *Acetivibrio* and ‘Unclassified *Clostridia*’, respectively, were about 10^6^*rrs* copies/μg DNA. The population of Ad-H1-14-1 was significantly higher in the Ad-C than in the other three fractions, while the population of Ad-H2-90-2 did not differ among all the fractions (Table 1) but tended (*P* < 0.1) to be lower in the Lq-H fraction than in the other three fractions. However, the distribution of the populations of Ad-H1-14-1 and Ad-H2-90-2 in sheep rumen [6] were not similar to that seen in the cow rumen. Ad-H1-14-1 accounted for more than 1% of total bacteria in all the fractions except the Lq-C fraction (Table 1). Because *Acetivibrio* only includes cellulolytic species such as *A. cellulolyticus* and *A. cellulosolvens *[19,20], Ad-H1-14-1 might represent an *Acetivibrio* bacterium that participates in fiber degradation in the rumen. Future studies targeting *Acetivibrio* can help further assess the importance of this genus to cellulose degradation in the rumen. The population of Ad-H1-75-1, which was assigned to ‘Unclassified *Clostridiales*’, was significantly higher in the Ad-H fraction than in the other three fractions (Table 1). Although the previous study [6] did not show significant difference in this uncultured bacterium among fractions, the population of Ad-H1-75-1 was numerically higher in sheep fed hay alone than in sheep fed hay plus corn. Thus, Ad-H1-75-1 might be a member of the biofilm adhering to feed particles and potentially involved in fiber degradation.

In our study, two measurements (*rrs* copy numbers vs. relative abundance) did not always result in the same significant differences among the four fractions. This discrepancy might be attributable to variable amounts of ruminal protozoal and fungal DNA contained in the metagenomic DNA that was used to normalize *rrs* copy numbers. More studies will need to be performed to verify this assumption.

Host genetics can have effects on the rumen microbiome and thus cattle of the same breed are typically used in the same study. In this study two different breeds were used and the number of animals analyzed were small. Nevertheless, the preliminary results of this study based on single sampling suggest that numerous uncultured bacteria are predominant in the rumen and may play an important role in ruminal fermentation. The function and ecological features of uncultured bacteria might be inferred from their population dynamics in cattle fed different diets. Alternatively, a reverse metagenomic approach [21,22], may be used to help isolate these uncultured bacteria. The metagenomic data recovered in previous studies of ruminal samples can be used to design selective media to grow uncultured bacteria through its metabolic reconstruction, while population data, as demonstrated in this study using specific real-time PCR, can be used to choose the dilutions as inoculum. Future studies may also determine to what extend animal breeds affect rumen microbiome.

## Conclusions

The populations of uncultured bacteria can be as great as those of major cultured bacteria. These uncultured bacteria are also ubiquitous in the rumen. Uncultured bacteria may play as an important role as some of the cultured bacteria, if not more. Comparative dynamic studies of uncultured bacteria in response to dietary treatments might help further reveal their ecological niche and roles in the rumen. Isolation and characterization of as-yet uncultured bacteria in the rumen would need to be attempted to define their functions and contribution to rumen functions.

## Abbreviations

*rrs*: 16S rRNA gene; Ad: Adherent; Lq, Liquid; H: Cattle fed hay alone; C: Corn silage and corn plus alfalfa hay; ANOVA: Analysis of variance.

## Competing interests

Both authors have no competing interests.

## Authors’ contributions

ZY and MK conducted this experiment and drafted the manuscript. ZY advised MK in performing the study and writing the manuscript for publication. All authors read and approved the final manuscript.
